# Effects of bisphenol A on root traits and rhizosphere bacteria: exploring the link between rhizosphere bacterial and root growth

**DOI:** 10.1186/s12866-025-04306-8

**Published:** 2025-08-29

**Authors:** Manli Yang, Shanningmei Zuo, Ahui Liu, Nana Zhong, Xueping Lu, Xun Liu, Xiasen Jiang, Tao Hu, Yuntong Liu, Xiaogang Ren, Kang Zhou, Chuansheng Wu

**Affiliations:** 1 Anhui Province Key Laboratory of Pollution Damage and Biological Control for Huaihe River Basin, Fuyang Normal University, Fuyang, 236037 Anhui China; 2 Jiangxi Provincial Key Laboratory of Carbon Neutrality and Ecosystem Carbon Sink, Lushan Botanical Garden, Jiangxi Province and Chinese Academy of Sciences, Jiujiang, 332900 China; 3 School of Chemical Biology and Environment, Yuxi Normal University, Yuxi, 653100 China

## Abstract

**Background:**

Bisphenol A (BPA), a widespread environmental pollutant, has been extensively studied for its effects on bacteria and plant, but its impact on rhizosphere bacterial communities and plant root traits is less understood. At the same time, the role of bacteria in helping plants resist adversity is widely recognized, but the relationship between BPA-induced with rhizosphere bacterial changes and root development is still unclear. Therefore, this study investigated the effects of varying BPA concentrations (1.5, 17.2, and 50 mg/L) on soybean root traits and rhizosphere bacterial communities, as well as the relationship between them.

**Result:**

The results revealed that BPA exposure significantly altered root traits, with root length, surface area, volume, and tip numbers being suppressed at 50 mg/L, while lower concentrations (1.5 and 17.2 mg/L) promoted root elongation and thickening. Bacterial community composition shifted notably, with Bacillota increasing and Pseudomonadota decreasing in relative abundance across all BPA treatments. Alpha diversity, measured by richness and Shannon_e indices, increased slightly at lower BPA concentrations, while beta diversity (Bray_Curtis and UniFrac) analysis showed significant differences, particularly at 50 mg/L. Community assembly processes (βNRI and βNTI) were dominated by deterministic mechanisms at lower BPA concentrations but shifted toward stochastic processes at 50 mg/L. Correlation analysis revealed significant relationships between bacterial community dynamics and root traits (Principal component PC1 and PC2), with alpha diversity indices influencing root traits represented by PC2 and beta diversity indices showing a negative correlation with PC1.

**Conclusions:**

BPA exposure not only alters root morphology and bacterial community structure but also highlights the intricate interplay between rhizosphere bacteria and plant roots under BPA stress. This study contributes to the theoretical understanding of plant–microbe interactions in contaminated environments and may inform future research on microbial involvement in plant stress responses.

**Supplementary Information:**

The online version contains supplementary material available at 10.1186/s12866-025-04306-8.

## Background

Bisphenol A (BPA) is a synthetic chemical widely used in the production of polycarbonate plastics, epoxy resins, and flame retardants, making it a ubiquitous environmental contaminant [35]. Due to its extensive industrial applications, BPA has permeated various environmental compartments, including soil, water, and sediments, raising significant concerns about its ecological and health consequences [1]. Evidence has been provided regarding the detrimental effects of BPA on plant physiology, particularly its disruption of root development [62]. Furthermore, BPA exposure has been shown to alter microbial communities in both aquatic and terrestrial ecosystems, leading to structural shifts and a reduction in microbial functionality [6, 40, 64]. These microbial changes are particularly noteworthy given the critical role of rhizosphere bacteria in plant health. Rhizosphere bacteria enhance root growth, facilitate nutrient uptake, and improve stress tolerance through mechanisms such as hormone regulation, exopolysaccharide production, and antioxidant defense [30]. The impact of BPA on rhizosphere bacteria may critically shape plant root responses to exposure, yet the relationship between BPA-induced microbial alterations and root development remains unclear. This knowledge gap underscores the need for further research to unravel the complex interactions among BPA contamination, microbial dynamics, and plant health, guiding strategies to mitigate ecological risks.

BPA has been shown to exert concentration-dependent effects on plant root systems. Low concentrations of BPA (e.g., 1.5 mg/L) have been reported to stimulate root elongation and increase root biomass in soybeans, whereas higher concentrations (e.g., 17.2 or 50 mg/L) markedly inhibit root growth and reduce surface area and biomass [29, 48, 57]. Similar inhibitory effects on root development have been observed in other crops such as tomatoes, lettuce, and *Arabidopsis thaliana* [2, 13]. High BPA levels have also been associated with morphological abnormalities including sparse lateral roots, reduced absorptive surface, and localized blackening [30, 38, 41, 58]. While previous studies have largely focused on root length, surface area, and biomass, the plant root system comprises a broader array of functional traits. Therefore, investigating the impact of BPA on comprehensive plant root traits is essential to deepen our understanding of its ecological consequences.

Previous studies have demonstrated that BPA exposure significantly alters bacterial community structure across various environments. For instance, BPA (40 mg/L) increased the *Gammaproteobacteria*, *Betaproteobacteria*, and *Bacteroidia* in anaerobic granular sludge [31], but reduced *Caldisericum* and *Coprothermobacter* in another sludge system with concentration of 5 mg/kg (He et al.2024). In soil, BPA promoted *Proteobacteria* in farmland [65], and enriched *Pseudomonas* and *Lutibacter* in rhizosphere [53]. BPA also influences bacterial diversity, but its effects on bacterial alpha diversity are inconsistent: BPA decreased diversity in sediments and soils [34, 63, 65], but slightly increased it in sludge [17]. Beyond alpha diversity, BPA concentration and exposure time also influence beta diversity, leading to changes in bacterial community structure and clear separation among treatments [20, 40, 63].Variation in beta diversity are usually regulated by community assembly processes [10, 59], yet how BPA drives these processes remains unclear. β nearest taxon index (βNTI) [47] and β network correlation index (βNRI) [39] are important indicators for evaluating the process of community assembly. Applying these indices can help disentangle the deterministic and stochastic forces shaping microbial responses to BPA.

Rhizosphere microbial communities play a crucial role in enhancing plant resilience under environmental stressors [12, 24, 28, 52]. Changes in the composition and assembly of bacterial communities can directly influence root traits such as root branching architecture, and nutrient uptake efficiency, thereby modulating plant adaptive responses to stress [16, 54]. In light of this, we hypothesize that rhizosphere bacterial communities are closely linked to root trait variation under BPA stress. We integrate microbial community assembly processes (βNTI/βNRI) with root phenotypic data to reveal how BPA alters microbial ecological dynamics and how these changes relate to root development. Specifically, we aim to: (1) evaluate the effects of BPA on root traits and rhizosphere bacteria, and (2) explore the associations between microbial assembly patterns and root trait responses.

## Materials and methods

### BPA treatment and experimental design

The greenhouse cultivation experiment was carried out in Fuyang Normal University (32°53′N, 115°46′E; Fuyang, China) from April 23, 2021, to May 17, 2021. During the cultivation period, the greenhouse conditions were maintained, with a daily lighting time of 14 h, an average temperature of 27.0 °C, and a mean humidity of 63%. The soybeans (*Glycine max*) used in the experiment were selected based on intact seed coats and uniform size and colour. To compare with previous research on the effects of BPA on plants, none BPA (CK (control group)) and three concentrations of BPA (1.5, 17.2, and 50 mg/L) were selected for this experiment [48, 61]. BPA was acquired from Shanghai Aladdin Biochemical Technology (> 99.0% (GC)). Each group consisted of thirty pots, and within each pot, twelve soybeans were planted at a uniform depth of 3 cm. According to prior research, it was determined that the half-life of the solution when applied to soil was seven days [66]. Consequently, to maintain a prolonged impact, a BPA solution with a half-concentration was administered weekly. Notably, owing to the retention of soil particles, BPA is retained on the soil surface, forming white particles. Therefore, the addition of BPA can only be used as a specific treatment in this study [57].

### Root collection and measurement

The duration of the pot culture experiment’s monitoring phase spanned approximately 25 days. Once the actual leaves of the soybean plants had emerged, seedlings exhibiting disparate growth rates were eliminated to ensure consistent growth within each treatment, with a total of 20 plants being retained per treatment. On May 17, 2021, samples were gathered because the intricate nature of the root system during the later seedling stage hindered the determination of root system traits. Intact soybean root systems were carefully collected and transferred into sterile 50 mL centrifuge tubes containing 20 mL of sterile phosphate-buffered saline (PBS; 6.33 g NaH2PO4·H2O, 16.5 g Na2HPO4·7H2O) supplemented with 200 µL of Silwet L-77. The tubes were vortexed at maximum speed for 15 s to separate loosely attached soil from the root surface [33]. The roots were then rinsed thoroughly with distilled water to remove residual soil. From the CK group and the BPA treatment groups of 1.5 mg/L, 17.2 mg/L, and 50 mg/L, a total of 9, 7, 7, and 8 intact soybean root systems were collected, respectively. After root cleaning, the root systems were scanned using an EPSON root scanner. The scanned images were analyzed with the WinRHIZO Pro 2019 root analysis system (Regent Instruments Inc., Quebec, Canada) to obtain morphological parameters, including total root length, surface area, volume, average diameter, and number of root tips.

### DNA extraction and sequencing analysis

Rhizosphere soil was collected by centrifuging the turbid suspension obtained from the root washing procedure described above. Total genomic DNA was extracted from these pellets using the HiPure Soil DNA Extraction Kit (Magen, Guangzhou, China) according to the manufacturer’s instructions. Five biological replicates per treatment were used for DNA extraction and sequencing. The V5-V7 region of the bacterial 16 S rRNA gene was amplified using the primer pair 799 F (AACMGGATTAGATACCCKG) and 1193R (ACGTCATCCCCACCTTCC). PCR was performed in two rounds with Q5^®^ High-Fidelity DNA Polymerase (New England Biolabs, USA). The first round included 30 cycles of amplification; the second round (12 cycles) was used to add Illumina adapters and sample-specific barcodes. Amplicons were purified using AMPure XP beads (Beckman Coulter, USA), quantified with a StepOnePlus Real-Time PCR System (Applied Biosystems), and pooled in equimolar amounts. Sequencing was conducted on the Illumina NovaSeq 6000 platform using 2 × 250 bp paired-end mode.

DNA sequencing data were analyzed via USEARCHv11 [11]. After the original data were merged, Primers were removed from the merged reads using the Perl script trim_primer_in fq.pl [46], and the redundant sequence was subsequently removed by quality control and UNOISE3 denoising was applied to obtain amplicon sequence variants (ASVs). The “otu_rare” command was used to normalize the feature table (ASV). Then all samples are based on the same reading of the minimum sample size to generate a clean ASV table for downstream analysis. The SILVA database SSU Ref NR 99 release 138.2 [45] annotated the bacterial sequence with a confidence threshold of 80%.

### Statistical analysis

Statistical analysis was performed using R (version 4.5.0) and Rstudio. For each response variable, including root morphological traits (e.g., length, surface area, volume) and bacterial metrics (e.g., richness, Shannon’s evenness, Bray_Curtis, UniFrac, βNRI, βNTI), the residual normality was first assessed using the Shapiro-Wilk test. Depending on the distribution of the residuals, generalized linear models (GLMs) were fitted using either a Gaussian distribution with identity link (for normally distributed data) or a Gamma distribution with log link (for non-normally distributed data). For all models, Type III sums of squares were used to assess the significance of treatment effects via the “Anova” function in the “car” package [14]. Post hoc pairwise comparisons among treatment groups were conducted using the “emmeans” package [26] with Sidak adjustment to control for multiple testing. Compact letter displays (CLDs) indicating significant differences were generated using the “multcomp” package [19].

Differences in root morphological traits under different BPA concentrations were visualized using boxplots. FactoMineR [25] was used to perform principal component analysis (PCA) on root length, surface area, volume, average diameter and number of tips, and the obtained two principal components (PC1, PC2) were analyzed and visualized. The alpha (e.g., richness, Shannon’s evenness index) and beta diversity (e.g., Bray-Curtis, UniFrac) analysis from the ASV table were completed using USEARCH v11. The bacterial community composition was analyzed at the phylum and genus levels, with the dominant phylum and certain genera further examined separately, using the same statistical methods as described earlier. To explore the effect of BPA on bacteria, partial least squares-discriminant analysis (PLS-DA) was used to visualize the ASV table [49]. To identify the taxa most responsible for group separation in the PLS-DA To identify the taxa most responsible for group separation in the PLS-DA model, we examined the absolute loading values of the first component (P1), and visualized in a biplot overlay. Group differences were statistically evaluated using PERMANOVA. Richness and Shannon’s evenness (Shannon_e) are commonly used to characterize the alpha diversity of microbial communities, reflecting species richness and distribution evenness, respectively. Bray_Curtis, UniFrac index, were used to characterize beta diversity. The β nearest taxon index (βNTI) and β network correlation index (βNRI) were calculated based on iCAMP (Ning et al. 2020) and picante [23] to explore the potential mechanisms of community assembly. For pairwise comparisons of βNTI and βNRI, we inferred homogeneous selection if values were < − 2 and variable selection if values were > + 2 (approximate values of ± 1.96 at two-sided 5% error level of each null distribution). If − 2 < values < + 2, stochasticity (e.g., dispersal and drift) may have a stronger influence than deterministic selection [47]. In order to further explore the relationship between the root traits and rhizosphere bacteria, the lm function was used to perform simple linear regression on the bacterial diversity, community construction process and the principal components of root traits PC1 and PC2 based on the ordinary least squares (OLS).

## Results

### Effects of BPA on soybean root traits

**Fig. 1:**
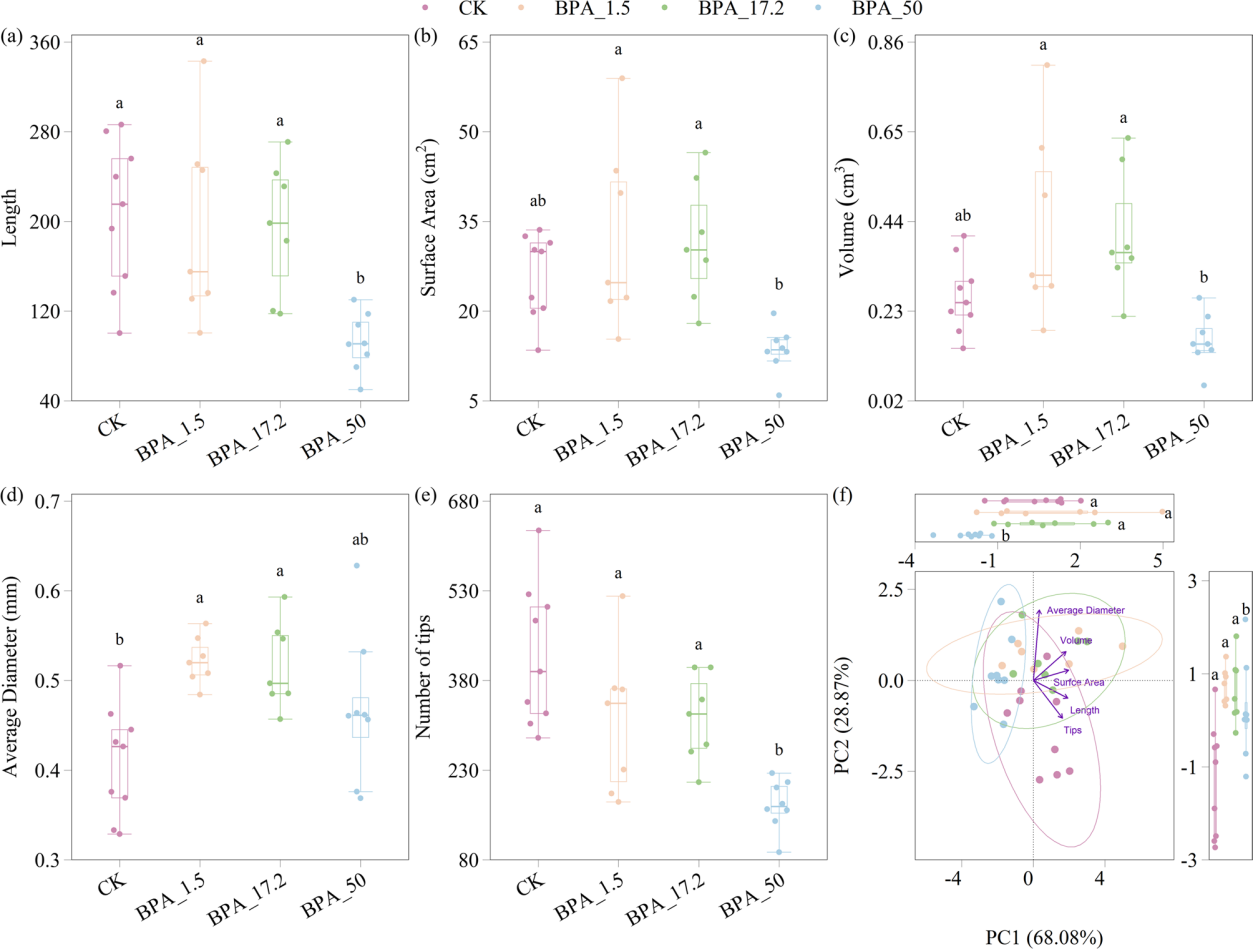
Response of root traits to different bisphenol A treatments. **a** Root length, **b** surface area, **c** volume, **d** average diameter, **e** root tip numbers. **f** Principal component analysis of all traits. The contribution rate of the first principal axis was 68.08%, and the contribution rate of the second principal axis was 28.87% and each point represented a sample, and the circle represents 95% confidence interval. Different lowercase letters indicate significant differences between treatments (*p* < 0.05) based on post hoc comparisons. Sample sizes for each group were as follows: CK (*n* = 9), BPA_1.5 (*n* = 7), BPA_17.2 (*n* = 7), BPA_50 (*n* = 8)

The impact of varying BPA concentrations on soybean root traits, including root length, surface area, volume, average diameter, and the number of root tips, was evaluated (Fig. 1 and S1). The root length was significantly reduced under 50 mg/L BPA treatment (*p* < 0.05) (Fig. 1a). No significant difference in root surface area was observed across the treatments, although a slight increase was seen at lower BPA concentrations (1.5 and 17.2 mg/L) and a decrease at the highest concentration (Fig. 1b). A similar trend was observed for root volume (Fig. 1c). The average diameter was significantly higher at 1.5 and 17.2 mg/L BPA concentrations (*p* < 0.05) (Fig. 1d). The number of root tips decreased across all BPA treatments compared CK, with reductions of 25.6%, 24.8%, and 59.2% at 1.5, 17.2 and 50 mg/L BPA concentrations, respectively (Fig. 1e).

Principal component analysis (PCA) was performed to assess variations in root morphological traits among different treatment groups. The first two principal components explained a total of 96.95% of the variance (PC1 = 68.08%, PC2 = 28.87%) (Fig. 1f). PCA biplot revealed clear group separation, particularly along PC1, indicating distinct root morphological responses under different treatments. Arrows represent the loading vectors of root traits, with length, tips, and surface area strongly contributing to the variation along PC1, while Average Diameter showed greater influence along PC2. High BPA concentrations (50 mg/L) significantly reduced PC1 (*p* < 0.05), while PC2 was significantly increased under all BPA treatments (*p* < 0.05). These findings indicate that BPA exposure, particularly at higher concentrations, significantly alters soybean root traits.

### Effects of BPA on rhizosphere bacteria

**Fig. 2:**
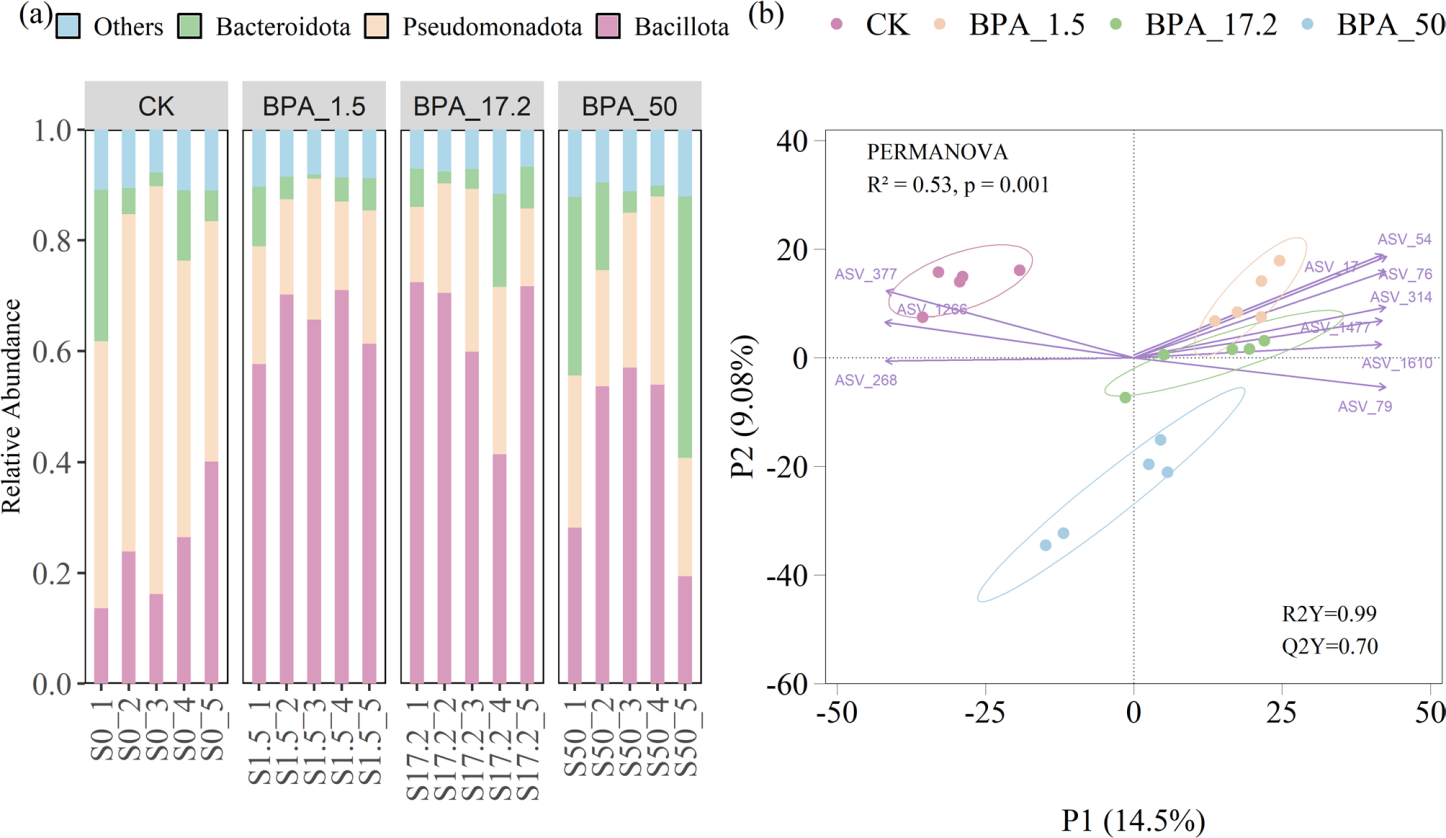
**a** Relative abundance of bacterial phylum in rhizosphere under different BPA exposure conditions. **b** Biplot of the PLS-DA model showing sample distribution and top contributing ASVs (based on loading scores). The model achieved R2Y = 0.99 and Q2Y = 0.70. Each point represented a sample, and the circle represents 95% confidence interval. Arrows indicate the direction and strength of association for the 10 most influential ASVs selected based on absolute loadings on the first components. Among the top 10 ASVs, seven (ASV_17, ASV_54, ASV_76, ASV_314, ASV_1477, ASV_1610, ASV_79) belonged to Bacillota, while the remaining three (ASV_377, ASV_1266, ASV_268) belonged to Pseudomonadota

The relative abundance of bacterial taxa was summarized at the phylum level across all treatment groups based on 16 S rRNA sequencing data (Fig. 2a). The rhizosphere bacterial community was dominated by the phylum Bacillota (average relative abundance 48.7%), Pseudomonadota (30.9%) and Bacteroidota (10.8%). Compared to CK, the relative abundance of Bacillota increased significantly across all the BPA treatments (*p* < 0.05) (Fig. S2a), while Pseudomonadota decreased significantly (*p* < 0.05) (Fig. S2b). Interestingly, Bacteroidota remained relatively stable, with a slight increase at 50 mg/L (Fig. S2c). PLS-DA confirmed significant differences in bacterial community structure between BPA treatments and CK (R²Y = 0.99, Q²Y = 0.70) (Fig. 2b). ASVs affiliated with Bacillota (e.g., ASV_54, ASV_76, ASV_314, ASV_17) exhibited high positive loadings along the P1 axis, contributing to the separation of BPA_1.5 and BPA_17.2 from the CK. Conversely, ASVs from Pseudomonadota (e.g., ASV_1266, ASV_268, ASV_377) showed negative loadings along P1, aligning with the CK. These loading patterns are consistent with the phylum-level shifts, where the relative abundance of Bacillota increased and Pseudomonadota decreased under BPA exposure.

**Fig. 3:**
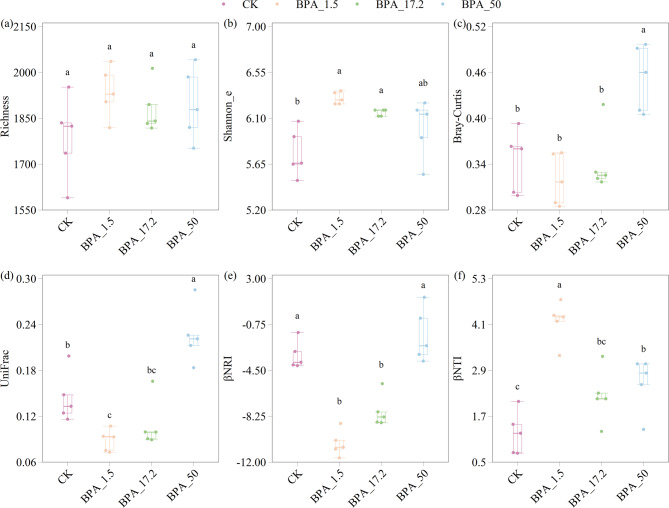
Ecological responses of bacterial communities to BPA exposure. **a** Richness and **b** Shannon_e (Shannon’s evenness) for alpha diversity assessment; **c** Bray_Curtis and **d** UniFrac demonstrate beta diversity; **e** βNRI, **f** βNTI represents the community assembly process. Superscript letters a-c indicate significance grouping. Each treatment group included five biological replicates (*n* = 5)

Alpha diversity was assessed using species richness and Shannon’s evenness (Shannon_e) index, which reflects the evenness of species distribution. Richness did not differ significantly among treatments (*p* > 0.05), though BPA treatments exhibited slight increases (8.3%, 5.2%, and 6.0% higher than CK) (Fig. 3a). The Shannon_e index increased significantly at 1.5 and 17.2 mg/L BPA concentrations (*p* > 0.05) (Fig. 3b). Beta diversity analysis revealed notable differences between treatments, particularly at high BPA concentrations (50 mg/L), which increased both Bray-Curtis and UniFrac distances (Fig. 3c, d). The distances for CK were greater than those for lower-concentration BPA treatments (1.5 and 17.2 mg/L). To explore bacterial community assembly under BPA stress, βNRI and βNTI values were assessed. Treatments with 1.5 and 17.2 mg/L BPA exhibited significantly higher |βNRI| values compared to the CK (*p* < 0.05), suggesting stronger influence of deterministic selection on phylogenetic composition. In contrast, the 50 mg/L treatment showed no significant difference from the control, implying a more stochastic assembly pattern (Fig. 3e). Similarly, all BPA treatments showed increased βNTI values relative to the CK, with values exceeding the |2| threshold, indicative of deterministic processes such as environmental filtering (Fig. 3f).

### Relationship between root traits and bacteria

**Fig. 4:**
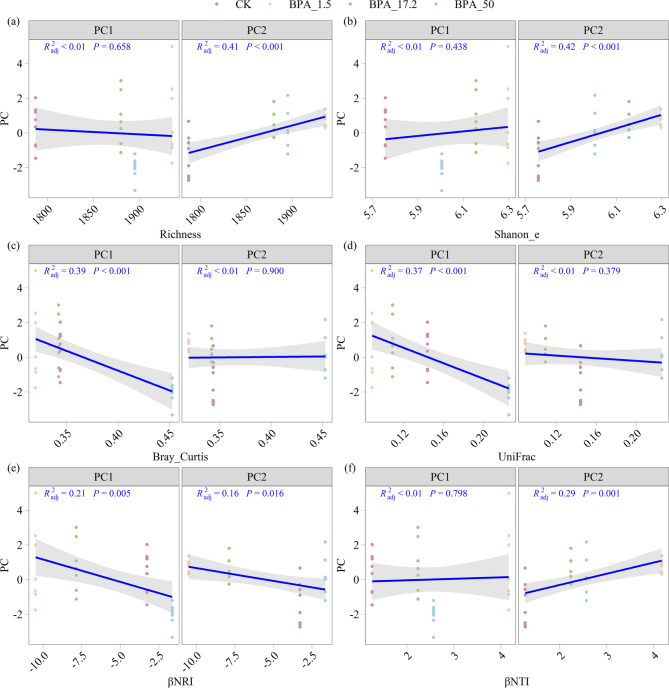
Correlation analysis between bacteria and principal components of root traits (PC1, PC2). (**a**-**b**) Correlation analysis of bacterial (**a**) richness and (**b**) Shannoon_e with principal components PC1 and PC2 of root traits; (**c**-**d**) Correlation analysis of (**c**) Bray_Cruist and (**d**) UniFrac with principal components PC1 and PC2; (**e**-**f**) Correlation analysis of (**e**) βBRI and (**f**) βNTI with principal components PC1 and PC2 Sample sizes for each group were as follows: CK (*n* = 9), BPA_1.5 (*n* = 7), BPA_17.2 (*n* = 7), BPA_50 (*n* = 8)

The alpha diversity indices (richness and Shannon_e) were not significantly associated with root traits represented by PC1 but showed strong correlations with PC2 (richness: R² = 0.41, Shannon_e: R² = 0.42; *p* < 0.001), together explaining up to 42% of the variation (Fig. 4a, b). In contrast, beta diversity indices (Bray-Curtis and UniFrac) were significantly negatively correlated with PC1 (Bray-Curtis: R^2^ = 0.39, UniFrac: R^2^ = 0.37, *p* < 0.001), but showed no significant association with PC2 (Fig. 4c, d). Furthermore, community assembly metrics (βNRI and βNTI) were significantly associated with PC2 (βNRI: R^2^ = 0.16, *p* = 0.016; βNTI: R^2^ = 0.29, *p* = 0.001), while βNRI also correlated with PC1 (R² = 0.21, *p* < 0.001) (Fig. 4e, f). Correlation analysis between bacterial components (P1, P2) and root traits (PC1, PC2) revealed that P1 was strongly associated with PC2 (R² = 0.45, *p* < 0.001), while P2 showed a significant correlation with PC1 (R² = 0.32, *p* < 0.001) (Fig. S5).

Regarding specific root traits, alpha diversity had the most significant effect on root average diameter and tips numbers (Fig. S3 a4-a5, b4-a5). Beta diversity, however, mainly affected other traits except average diameter (Fig. S3 c, d). Additionally, Community assembly metrics (βNRI) significantly influenced all traits, except for the number of root tips (Fig. S3 e), while βNTI was only significantly correlated with average diameter (explaining 23% of the variance) (Fig. S3 f).

## Discussion

### BPA’s concentration-dependent effects on root growth

This study revealed that BPA exerts significant concentration-dependent effects on soybean root systems (Fig. 1). The results demonstrate that high-concentration BPA (50 mg/L) inhibits multiple morphological parameters of roots (length, surface area, and tips), with its adverse effects aligning with prior studies [13, 58, 62]. Notably, at lower concentrations (1.5 and 17.2 mg/L), multiple traits of roots were promoted, and the promotion effect of root average diameter was the most significant (Fig. 1d). This low-concentration promotion effect was repeatedly verified in the many culture systems [38, 56]. The concentration-dependent modulation of root traits by BPA has been corroborated across multiple cultivation experiments [30, 41, 48, 58], while the intermediate concentration (17.2 mg/L) identified in this study exhibited a distinct response—unlike the general inhibitory effects observed in most hydroponic studies [29, 48], parameters such as root length and volume showed slight increases in our experiments (Fig. 1a, c). This discrepancy may be attributed to the activation of BPA-degrading microbial communities in soil, whose degradation activity at specific concentration thresholds likely alleviates BPA-induced stress [34].

The generation of this concentration gradient effect cannot ignore the metabolic transformation of BPA in plants and the molecular response triggered by BPA. BPA can be converted into plant-available precursor substances and regulates the activity of key enzymes in chlorophyll synthesis [22], significantly affecting plant physiological processes. Appropriate application can increase chlorophyll content [44], while enhancing auxin synthesis (30). Therefore, at concentrations that do not cause absolute plant damage, low-dose BPA can improve photosynthetic capacity and growth potential. However, although high concentrations of BPA are also convertible, the energy metabolism disorder [61] and oxidative damage [67] caused by it have crossed the toxicological threshold, resulting in the positive effect being masked. Our findings demonstrate that BPA consistently shows adverse effects on root tips (Fig. 1), with intensity escalating at higher concentrations. These impacts suggest BPA may interfere with root differentiation processes. Transcriptome data reveals that high-concentration BPA suppresses gene expression related to lateral root formation and xylem development [57], thereby negatively affecting nutrient acquisition. Concurrently, it upregulates stress-resistance gene expression [51, 57], collectively shaping root phenotypic characteristics.

### BPA-induced shifts in rhizosphere bacterial communities

BPA exposure significantly altered the composition and structure of rhizosphere bacterial communities: control group showed average abundances of 24.1% Bacillota (formerly Firmicute) and 55.2% Pseudomonadota (formerly Proteobacteria), whereas 1.5 mg/L BPA treatment elevated Bacillota to 65.2% and reduced Pseudomonadota to 20.8% (Fig. 2a). To validate compositional changes, PLS-DA showed clear separation between BPA-treated and control groups (Fig. 2b). The ASVs contributing most to this separation were consistent with the observed phylum-level trends. Specifically, Bacillota-affiliated ASVs (e.g., ASV_54, ASV_76) increased in abundance and contributed positively along the P1 axis, suggesting a potential role in stress adaptation or BPA degradation. In contrast, Pseudomonadota-associated ASVs (e.g., ASV_1266, ASV_268) were negatively loaded and diminished under BPA, possibly indicating their sensitivity to this compound. These compositional shifts likely reflect the functional strategies of key bacterial phyla in response to BPA stress. Although Pseudomonadota are widely known for their BPA-degrading capacity [20, 65], their abundance declined with BPA addition, possibly due to toxicity exceeding microbial tolerance over time [6]. In contrast, Bacillota increased, likely due to spore-forming traits that enhance resistance [31]. Notably, *Bacillus*, known BPA degraders, were enriched under BPA exposure (Fig. S4), indicating potential involvement in BPA biodegradation [27]. Bacteroidota maintained stable abundance across all BPA levels, consistent with previous findings suggesting their role as neutral responders to environmental stress [60]. Although not directly enriched, their ecological resilience may contribute to community homeostasis under chemical stress [42].

BPA-driven community restructuring further caused diversity fluctuations. Initial increases in Shannon_e were observed at low BPA concentrations, indicating enhanced community evenness. But Shannon_e stagnated at 50 mg/L BPA (Fig. 3b). The dose effect of this bacterium on BPA was verified in multiple experiments [20, 53, 63]. These changes may be the result of stress selection: low BPA (1.5 and 17.2 mg/L) activated key metabolic pathways in tolerant taxa [7, 68], leading to increased evenness and thus elevated Shannon_e index. This phenomenon may resemble a hormesis-like effect, where mild environmental stress stimulates microbial diversity or enhances community balance [32]. However, at higher BPA concentrations, the strong selective pressure caused the extinction of sensitive taxa, rapid release of niche space, and a community turnover dominated by a few tolerant species [9, 37]. The antagonism between colonization efficiency and ecological drift of newly recruited taxa eventually led to the stagnation of alpha diversity at 50 mg/L BPA.

A non-linear dose-dependent analysis of BPA exposure revealed distinct impacts on microbial beta diversity and the balance between deterministic and stochastic community assembly processes (Fig. 3). Under low BPA concentrations, beta diversity remains at reduced levels, where the competitive advantage of evolutionarily conservative species forms community filtering screens, allowing deterministic processes to dominate community assembly [39]. When concentrations exceed the threshold, beta diversity indices (Bray-Curtis, UniFrac) increase significantly, suggesting enhanced community differentiation. This shift may reflect increased community instability, intensified niche differentiation, and the emergence of rare/opportunistic taxa under high-stress conditions [18]. These observations are consistent with previous studies showing that beta diversity can reflect the balance between deterministic and stochastic community assembly in response to environmental stress [36]. This is further supported by the shift in βNTI and βNRI values: at low BPA concentrations, an increase in βNTI (> + 2) indicates that deterministic processes dominated community assembly, with BPA acting as an environmental filter that directed the community along a deterministic trajectory [8]. In contrast, at 50 mg/L BPA, βNRI values approached zero, suggesting a shift towards stochastic assembly processes. This pattern reflects a disruption of community equilibrium under high BPA stress, allowing random mechanisms such as ecological drift and neutral colonization to prevail [37]. This dose-dependent transition—from deterministic to stochastic community assembly—highlights BPA’s dual role in shaping the rhizosphere microbiome: low concentrations stabilize communities via functional enrichment and niche conservatism [15], while high concentrations destabilize networks, amplify stochastic niche allocation, and lead to unpredictable community structures [36, 64]. These trends are consistent with an extended interpretation of the Stress Gradient Hypothesis, which posits that as environmental stress increases, deterministic interactions weaken, giving way to more stochastic and unpredictable community assembly [4].

### Interplay between rhizosphere bacteria and root growth

The interaction between rhizosphere bacterial communities and root growth under BPA exposure exhibited a dose-dependent regulatory pattern (Fig. 4). BPA stress significantly inhibited the abundance of Pseudomonadota (Fig. S2), leading to reduced symbiotic nitrogen fixation capacity in its key functional group (rhizobia), impaired soybean root tip differentiation, and decreased nitrogen fixation efficiency [56]. However, lower BPA concentrations (1.5 and 17.2 mg/L) triggered a plant-microbe coordinated defense mechanism: the relative abundances of *Bacillus* increased under BPA treatment (Fig. S4), converting BPA molecules into metabolic intermediates and activating multiple pathways to alleviate nutrient absorption inhibition [27]. These plant growth-promoting bacteria enhanced stress tolerance by regulating plant genes (e.g., ABA biosynthesis genes), increasing osmotic protectants (e.g., proline) [5, 57], and activating antioxidant enzyme systems (SOD, APX) to mitigate ROS damage [12]. The positive correlation between microbial Shannon_e and root principal component PC2 (R² = 0.42) (Fig. 4a) further confirmed the systemic plant-microbe regulatory mechanism. For example, *Bacillus subtilis* improves nutrient availability, modulates phytohormone homeostasis, and alleviates abiotic stress [5, 55]. This directional enrichment of functional bacterial communities originated from bidirectional plant-microbe signaling: BPA-induced root exudates selectively recruited aromatic-degrading bacteria [7], which accelerated biotransformation via upregulated the expression of resistance gene [50].

When BPA concentrations exceeded 50 mg/L, functional thresholds of microbial communities led to mutualism collapse. At this stage, toxic metabolites shifted community assembly from deterministic selection to stochastic drift [43], causing ecological niche mismatch. The further decline of Pseudomonadota reduced symbiotic nitrogen fixation efficiency [56]. Meanwhile diminished of carboxylates (e.g., citrate, malate) in root exudates impaired bacterial colonization in root tip protection zones [3]. Such multi-level dysregulation disrupted plant-microbe energy exchange balance—roots prioritized resource allocation to stress defense over growth, resulting in growth stagnation. This phenomenon aligns with the defense-growth trade-off in stress adaptation [21], where plants under high contamination preferentially allocate resources to stress resistance. The dose-dependent pattern reveals the dynamic interplay between rhizosphere microbiota and root architecture: low BPA concentrations activate plant intrinsic resistance via detoxification-nutrition coupling by functional bacteria, whereas metabolic bottlenecks and signaling interference under high-concentration stress lead to collapse of plant-microbe mutualistic networks.

## Conclusion

This study demonstrates that BPA exposure has dose-dependent effects on both soybean root traits and rhizosphere bacterial communities. Higher BPA concentrations inhibit root growth and induce significant shifts in bacterial community structure and diversity. The interplay between plant roots and rhizosphere bacteria plays a critical role in mediating the plant’s response to BPA, with deterministic processes dominating at low concentrations and stochastic processes prevailing at high concentrations. These findings improve our understanding of how environmental contaminants like BPA shape plant–microbe interactions and suggest that microbiome-level responses may inform future research into mitigating pollutant-induced stress in crops. While our findings highlight important trends, further research is required to experimentally validate the functional contributions of microbial communities to plant adaptation under BPA stress.

## Supplementary Information

Supplementary Material 1.

## Data Availability

All data generated or analyzed during this study are included in this published article and its supplementary information files. The raw sequencing data have been deposited in the NCBI SRA under BioProject accession number PRJNA1251285, with individual runs accessible under accession numbers SRR33171570 to SRR33171589.
