# Supplementary material for: Effects of bisphenol A on root traits and rhizosphere bacteria: exploring the link between rhizosphere bacterial and root growth
Source: BMC Microbiol. 2025 Aug 29;25:560. doi: 10.1186/s12866-025-04306-8 (PMC12395689; doi:10.1186/s12866-025-04306-8)
Supplement: Supplementary file 1 — Supplementary Material 1. [file 12866_2025_4306_MOESM1_ESM.docx]

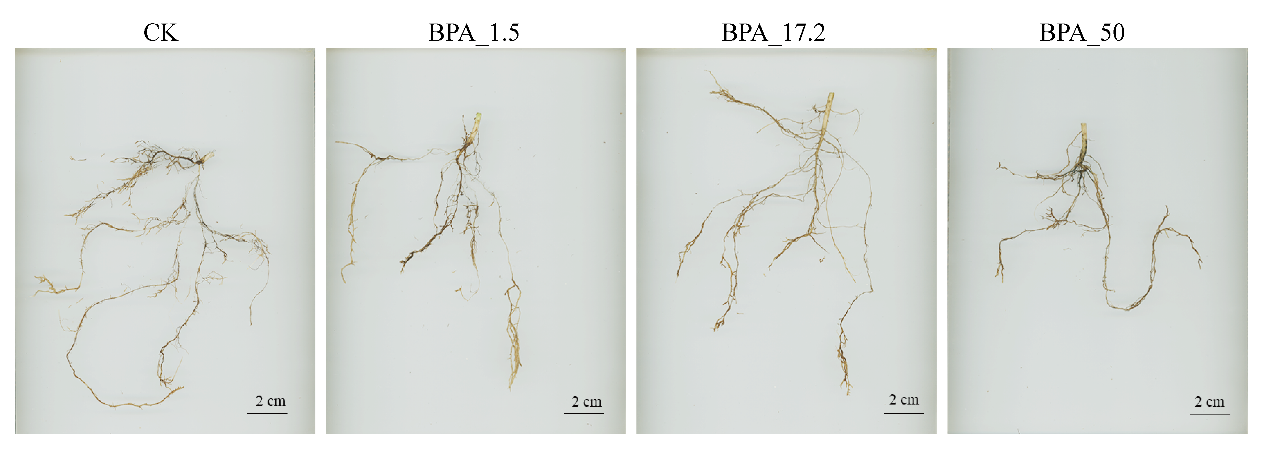


Figure S1. Root scanning under four BPA treatments, the root morphology shows the root traits of CK, 1.5 mg/L BPA, 17.2 mg/L BPA, and 50 mg/L BPA.


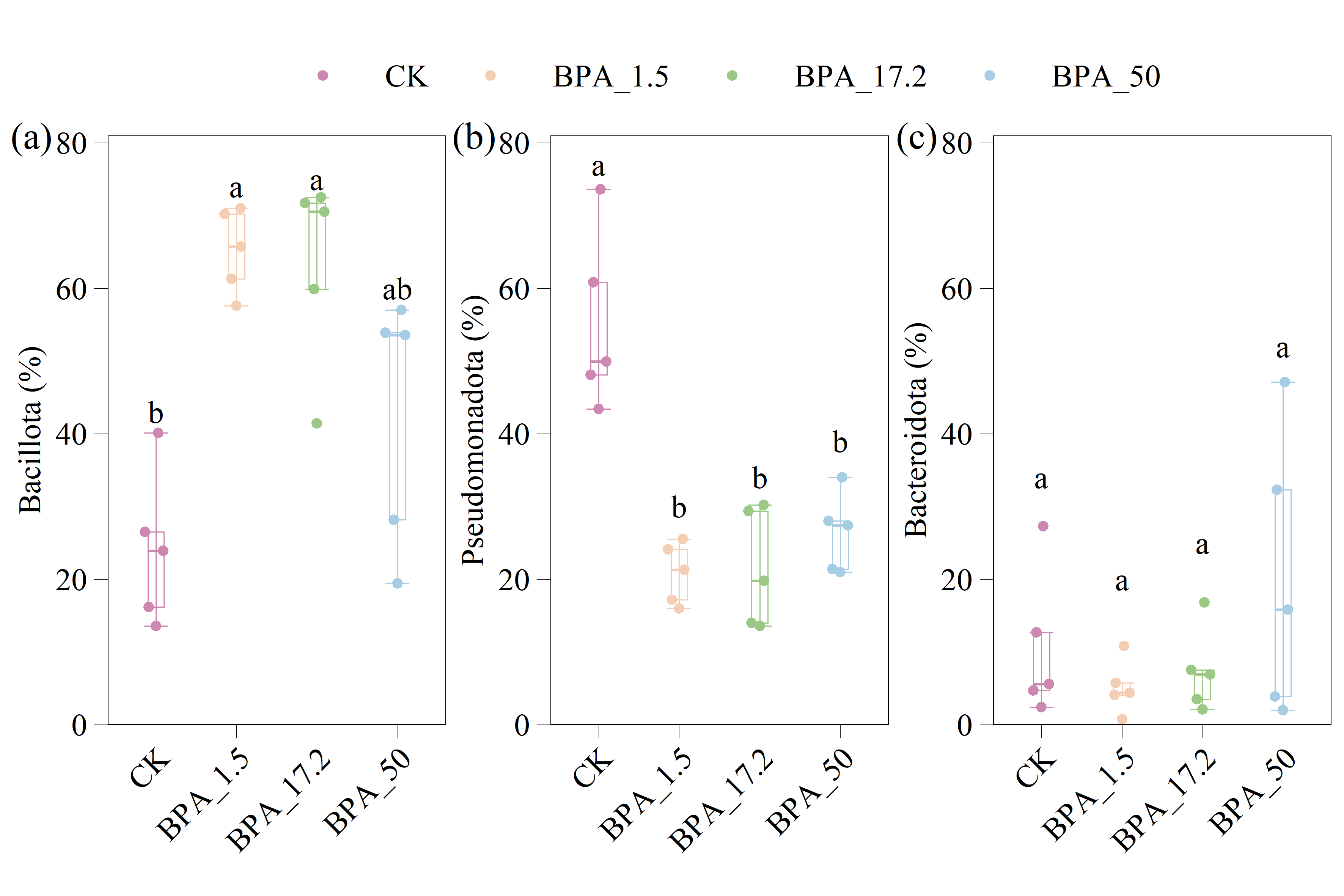


Figure S2. Relative abundance of (a) Bacillota, (b) Pseudomonadota, (c) Bacteroidota under different BPA concentrations (CK, 1.5, 17.2 and 50 mg/L). Different lowercase letters indicate significant differences between treatments (*p* < 0.05) based on post hoc comparisons. Each treatment group included five biological replicates (n = 5).


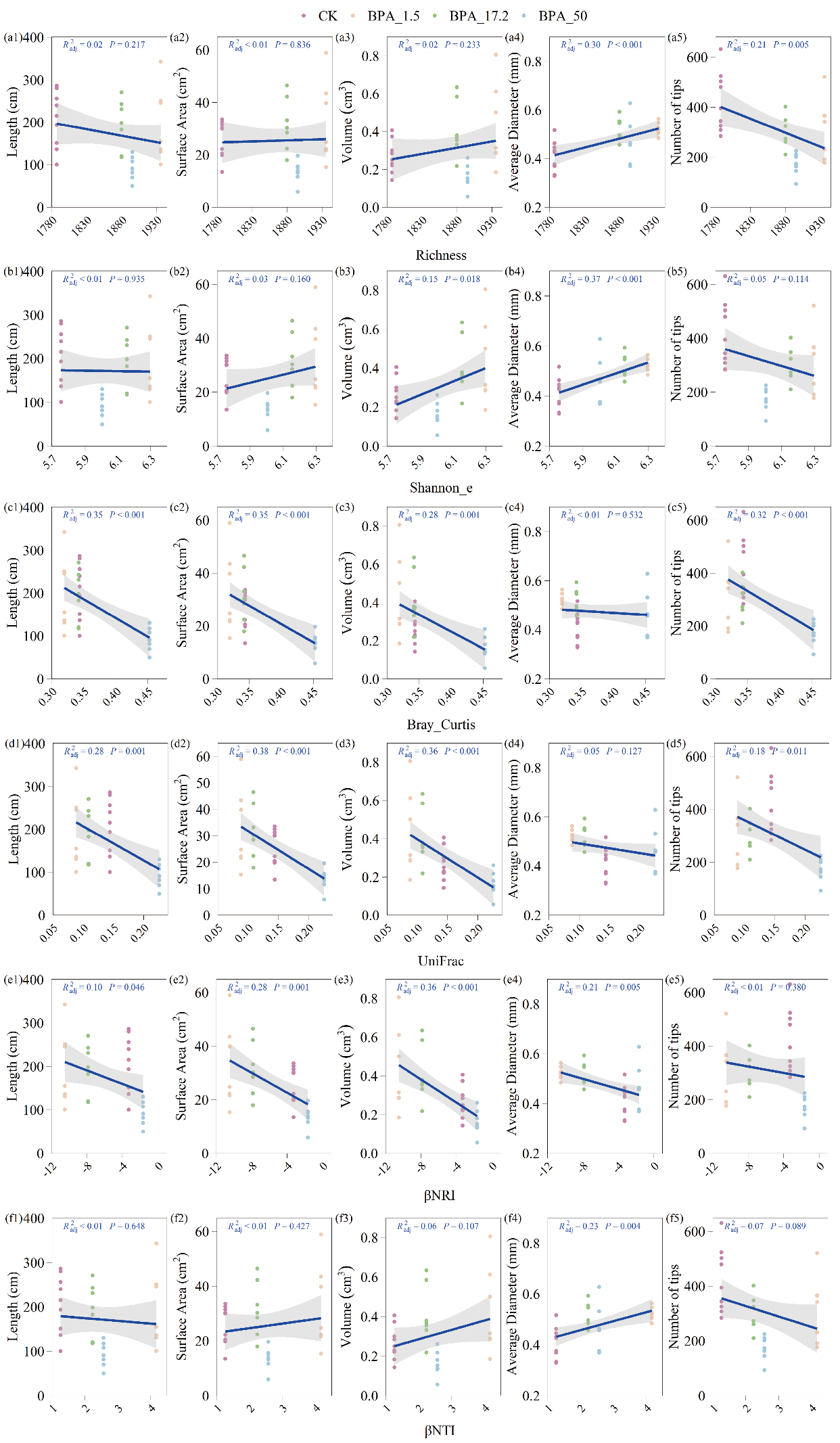


Figure S3. Linear regression between microbial community indices and root morphological traits (Length, Surface Area, Root Volume, Average Diameter, Tips).
(a1–a5) and (b1–b5) alpha diversity (richness and Shannon_e). Both and Shannon_e showed significant positive with average diameter (R² = 0.30 and 0.37, respectively, *p* < 0.001), and richness have negative correlations with number of tips (R² = 0.21, *p* = 0.005), while Shannon_e associations with root volume was significant positive (R² = 0.15, *p* = 0.018).
(c1–c5) and (d1–d5) beta diversity (Bray-Curtis and UniFrac). Both exhibited significant negative correlations with root length (R² = 0.35 and 0.28), surface area (R² = 0.35 and 0.38), and root volume (R² = 0.28 and 0.36), but had little to no correlation with average diameter. Tip number was also negatively associated with both indices (*p* < 0.001).
(e1–e5) and (f1–f5) community assembly metrics (βNRI and βNTI). βNRI showed significant negative correlations with surface area (R² = 0.28), root volume (R² = 0.36), average diameter (R² = 0.21). While βNTI showed weaker correlations overall, only average diameter (R² = 0.23, *p* = 0.004) was significantly associated with βNTI.

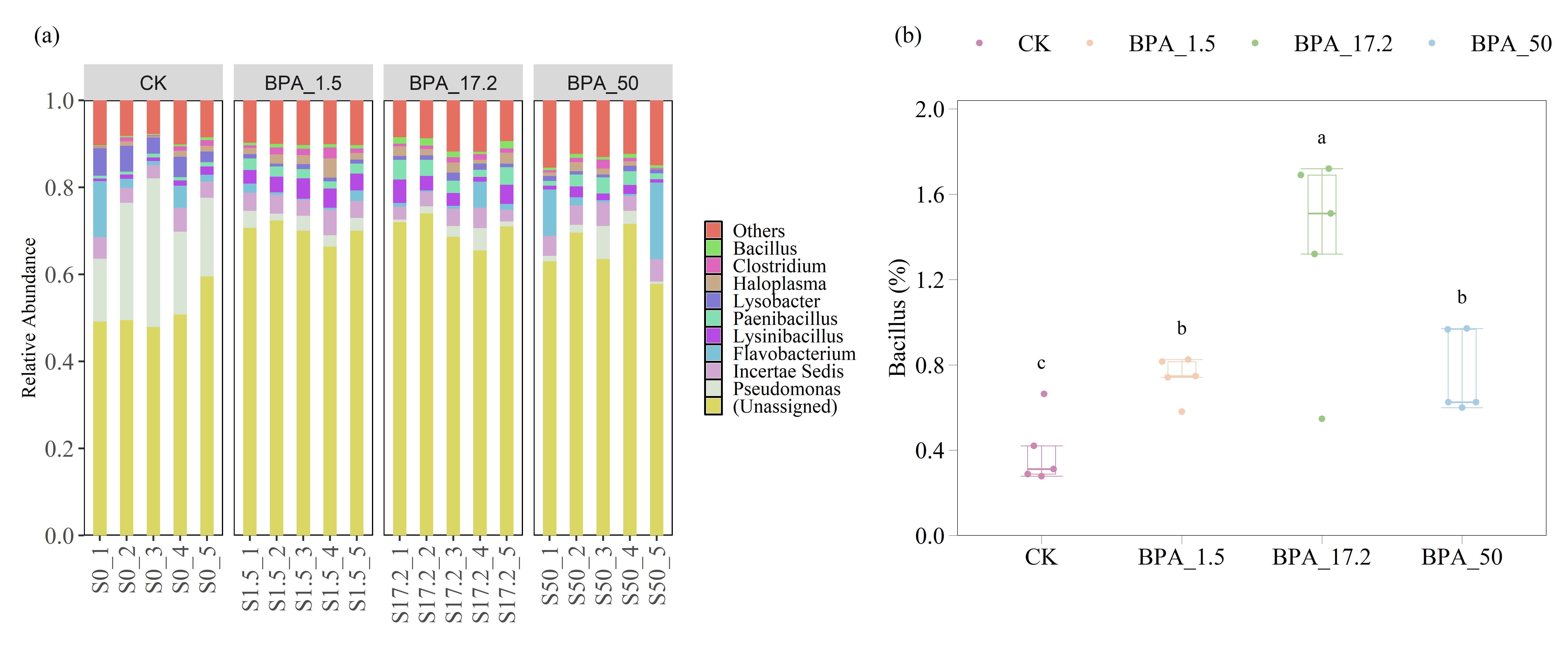


Figure S4. (a) The relative abundance of rhizosphere bacteria (top 10) at the genus level under four BPA concentrations. (b) *Bacillus* under different BPA concentrations (CK, 1.5, 17.2 and 50 mg/L). Different lowercase letters indicate significant differences between treatments (*p* < 0.05) based on post hoc comparisons. Each treatment group included five biological replicates (n = 5).


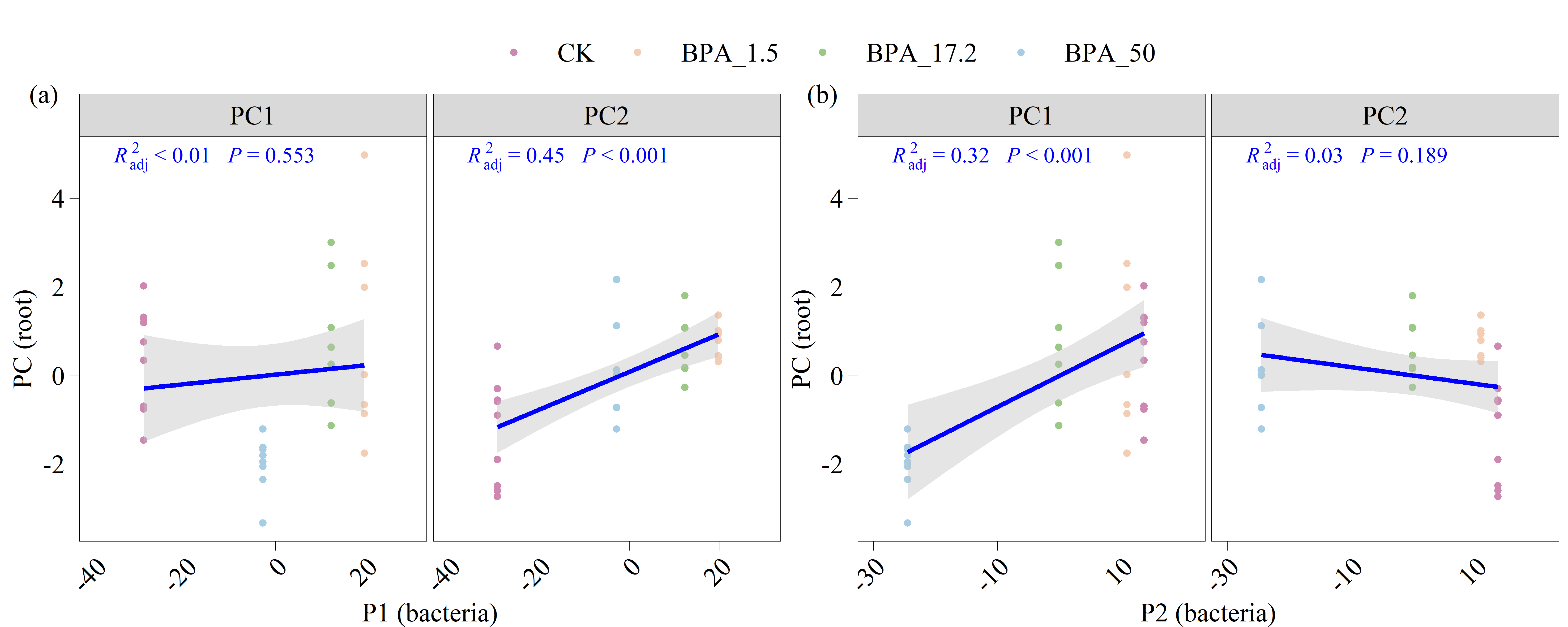


Figure S5. Correlation analysis of bacteria (P1, P2) and root traits (PC1, PC2).
